# [Corrigendum] MicroRNA-1 promotes cartilage matrix synthesis and regulates chondrocyte differentiation via post-​transcriptional suppression of Ihh expression 

**DOI:** 10.3892/mmr.2026.13911

**Published:** 2026-05-20

**Authors:** Taoyu Chen, Xianda Che, Pengfei Han, Jiangong Lu, Chunfang Wang, Bin Liang, Ziqi Hou, Xiaochun Wei, Lei Wei, Pengcui Li

Mol Med Rep 22: 2404–2414, 2020; DOI: 10.3892/mmr.2020.11296

Following the publication of this paper, it was drawn to the Editor's attention by a concerned reader that, regarding the EdU cell proliferation assay data shown in Fig. 1C on p. 2408, the miR-1 and Control data panels showed a small overlapping section, suggesting that data which were intended to show the results of differently performed experiments had apparently been derived from the same original source.

The authors were contacted by the Editorial Office to offer an explanation for this apparent anomaly in the presentation of the data in this paper, and they have replied to explain that the data panels representing the Control experiments were inadvertently selected incorrectly for this figure. The revised version of Fig. 1, now containing the correct data for the Control experiments in Fig. 1C, is shown on the next page. The authors confirm that this error did not affect the overall conclusions reported in this study. All the authors agree with the publication of this corrigendum, and the authors are grateful to the Editor of *Molecular Medicine Reports* for allowing them the opportunity to publish this. The authors regret that these errors were included in the paper, and also apologize to the readership for any inconvenience caused.

## Figures and Tables

**Figure 1.**
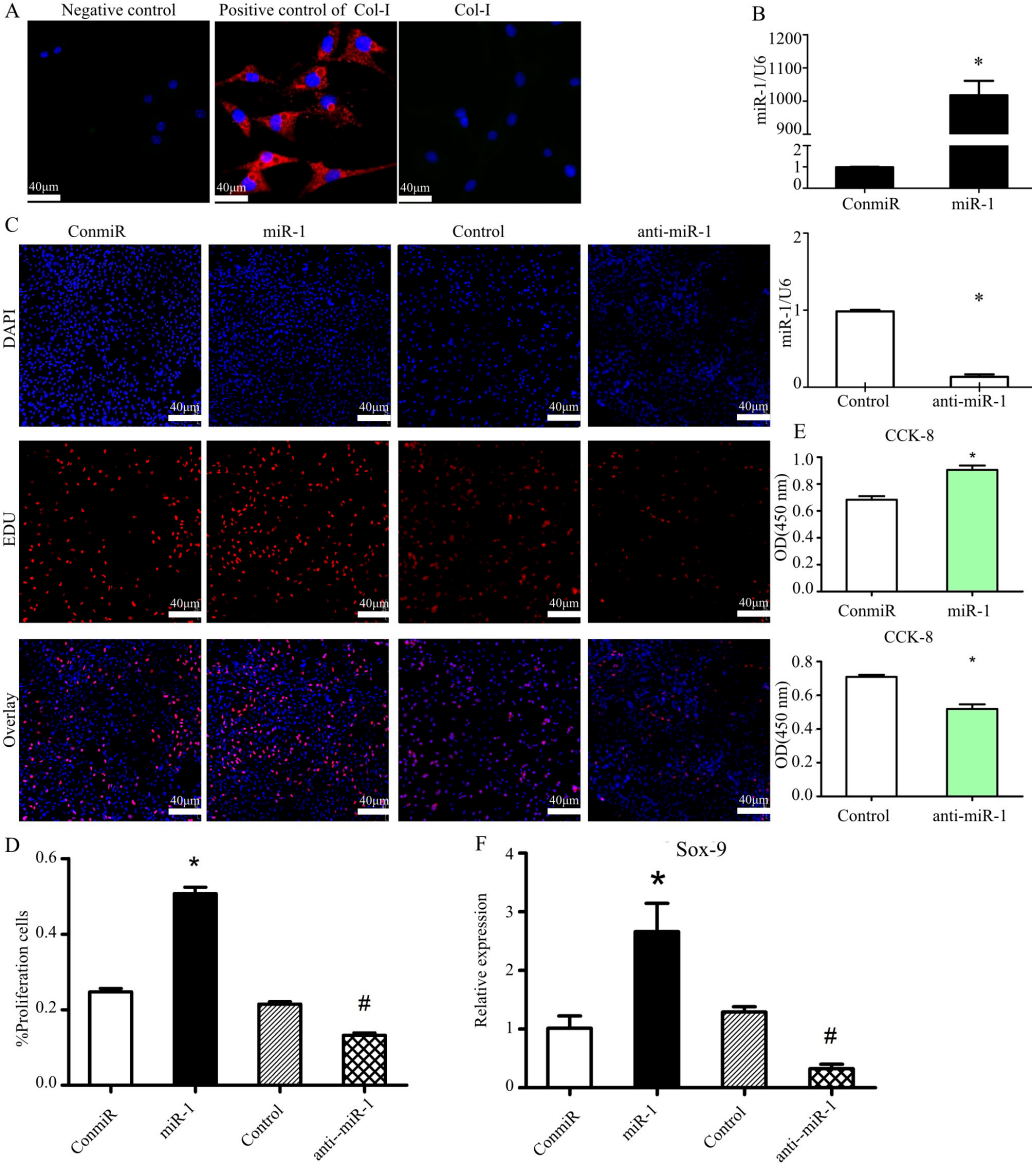
miR-1 promotes the proliferation of mouse thorax chondrocytes. (A) Expression of Col-I in mouse thorax chondrocytes was assessed using immunofluorescence staining. Mouse fibroblasts were used as the positive control for Col-I staining; magnification, ×40; scale bars, 40 µm. (B) RT-qPCR results showed that transfection of the miR-1 mimic (40 pM) increased miR-1 levels, whereas transfection of the anti-miR-1 decreased its expression, compared with the transfection of a negative control mimic (ConmiR) or a control miRNA inhibitor (Control) in the mouse sterna chondrocytes at 24 h post-transfection. n=3 for each group; *P<0.05 vs. ConmiR or Control. (C and D) Cell growth was measured by EdU cell proliferation staining 48 h after mouse sterna chondrocytes were transfected with the miR-1 mimic (miR-1) or negative control mimic (ConmiR), and miR-1 inhibitor (anti-miR-1) or a control miRNA inhibitor (Control) at 120 nM, respectively; (C) images show the staining for EdU and DAPI, and (D) bar graph data summarizes the percentage of EdU-proliferating cells in 5 view fields per group; miR-1 stimulates chondrocyte proliferation. Scale bars, 40 µm; *P<0.05 vs. ConmiR; #P<0.05 vs. Control. (E) Transfection of miR-1 enhanced mouse thorax chondrocyte proliferation, while transfection of anti-miR-1 inhibited proliferation, as measured by the CCK-8 cell proliferation assay. n=5 for each group; *P<0.05 vs. ConmiR or Control. (F) miR-1 increased Sox-9 mRNA levels, a marker for chondrocyte proliferation. Sox-9 mRNA levels in mouse sterna chondrocytes transfected with the miR-1 mimic (miR-1), or control miRNA mimic (conmiR), and inhibitor (anti-miR-1) or control miRNA inhibitor (Control) were quantified by RT-qPCR at 24 h post transfection. n=3 for each group; *P<0.05 vs. ConmiR; #P<0.05 vs. Control. Col, collagen; Sox9, SRY-box transcription factor 9; RT-qPCR, reverse transcription-quantitative PCR; miR/miRNA, microRNA.

